# Long-Term Suppressive Therapeutic-Drug-Monitoring-Guided Dalbavancin Therapy for Cardiovascular Prosthetic Infections

**DOI:** 10.3390/antibiotics12111639

**Published:** 2023-11-19

**Authors:** Altea Gallerani, Milo Gatti, Andrea Bedini, Stefania Casolari, Gabriella Orlando, Cinzia Puzzolante, Erica Franceschini, Marianna Menozzi, Antonella Santoro, Nicole Barp, Sara Volpi, Alessandra Soffritti, Federico Pea, Cristina Mussini, Marianna Meschiari

**Affiliations:** 1 Department of Infectious Diseases, Azienda Ospedaliero-Universitaria of Modena, 41124 Modena, Italy; andreabedini@yahoo.com (A.B.); casolaristefania@hotmail.com (S.C.); antonella.santoro7@gmail.com (A.S.); nicole.barp94@gmail.com (N.B.); 307830@studenti.unimore.it (A.S.);; 2 Clinical Pharmacology Unit, Department for Integrated Infectious Risk Management, IRCCS Azienda Ospedaliero Universitaria di Bologna, 40138 Bologna, Italy; milo.gatti2@unibo.it (M.G.); federico.pea@unibo.it (F.P.); 3 Department of Medical and Surgical Sciences, Alma Mater Studiorum, University of Bologna, 40138 Bologna, Italy

**Keywords:** dalbavancin, prosthetic valve endocarditis, cardiac implantable electronic devices infections, prosthetic vascular graft infections, therapeutic drug monitoring

## Abstract

Dalbavancin represents a promising treatment for cardiovascular prosthetic infections due to its prolonged half-life, bactericidal activity, large spectrum of activity, and excellent biofilm penetration. However, the use of dalbavancin in this setting is limited, and only a few cases have performed therapeutic drug monitoring (TDM) analysis to optimize dosage in suppressive treatments longer than 4 weeks. Our retrospective case series reports the use of dalbavancin in a small cohort of patients with cardiovascular prosthetic infections (cardiac implantable electronic device infections (CEDIs), prosthetic valve endocarditis (PVE), prosthetic vascular graft infections (PVGIs)) treated with dalbavancin as sequential therapy. From May 2019 to May 2023, 14 patients were included: eight cases of PVE (57.1%), seven cases of PVGI (50%), three cases of CEDI (21.4%), and four cases with overlap of infection sites (28.6%). The main pathogen was Staphylococcus aureus (35.7%). Prosthesis replacement was obtained in four patients (28.6%). The median time between symptom onset and the end of treatment was 15 weeks (IQR 7–53), with a median duration of dalbavancin therapy of 8 weeks (IQR 1 to 45 weeks) and 3.5 doses per patient. Among patients managed with TDM-guided strategy, dalbavancin infusion intervals ranged from 4 to 9 weeks. The median length of follow-up was 65 weeks (IQR 23 to 144 weeks). Clinical success was achieved in 10 cases (76.9%); all clinical failures occurred in patients with the implant retained. Among patients monitored by TDM, clinical success was 87.5% vs. 60% in patients treated without TDM. Because of pharmacokinetic individual variability, dalbavancin TDM-guided administration could improve clinical outcomes by individualizing dosing and selecting dosing intervals. This case series seems to suggest a promising role of long-term suppressive dalbavancin treatment for difficult-to-treat cardiovascular prosthesis infection, also with limited surgical indications.

## 1. Introduction

Dalbavancin is a long-acting parenteral lipoglycopeptide, a semisynthetic derivative of teicoplanin. It is characterized by a large spectrum of action against Gram-positive bacteria, such as *Staphylococcus aureus* (including methicillin-resistant *S. aureus*, but also strains with intermediate sensitivity to vancomycin), *Streptococcus agalactiae*, *Streptococcus pyogenes*, *Streptococcus anginosus*, vancomycin-sensitive *Enterococcus faecium*, and *Enterococcus faecalis*; it is poorly active against vancomycin-resistant *S. aureus* (VRSA), and it is not active against vancomycin-resistant enterococci with the Van A phenotype [1,2].

According to its pharmacokinetic (PK) properties, dalbavancin shows a long elimination half-life (i.e., 14 days), coupled with a total protein binding of 93% [3]. Its clearance is predominantly renal, with no active metabolites and no influence by other medications inducing or inhibiting cytochrome P450 [4]. It is associated with good tissue penetration [3,5], including staphylococcal biofilms, making this antibiotic a promising agent to treat biofilm-mediated infections [6].

Dalbavancin is currently approved by the Food and Drug Administration (FDA) and the European Medicines Agency (EMA) only for acute bacterial skin and skin structure infections (ABSSSIs), with a standard dosage of 1500 mg in a single infusion or 1000 mg at day one followed by 500 mg at day eight [7]. Despite this limited indication coming from randomized controlled trials, several successful real-life experiences with dalbavancin for infections outside ABSSSIs have been increasingly reported in recent years; after osteoarticular infections, the greatest use of dalbavancin is for cardiovascular infections [8].

Cardiovascular infections are typically caused by Gram-positive pathogens, are associated with a significant risk of mortality, and usually require long-term antibiotic therapy as well as surgical revision [9]. A recently published review addressing the role of sequential dalbavancin in infective endocarditis found an overall clinical efficacy of around 90%, with a good safety profile despite widely variable dosages. The median duration of dalbavancin therapy was 6 weeks, with an average of three doses of drugs; however, only 2 patients out of 170 received dalbavancin as chronic suppressive therapy for a total of 6 months. This can be partially explained by the low rate of prosthetic valve endocarditis (PVE) (27%) and cardiac implantable electronic device infections (CEDIs) (12%). Prosthetic vascular graft infections (PVGIs) were completely excluded [10]. Recent evidence reported that therapeutic drug monitoring (TDM) may be a useful tool for estimating the optimal duration of dalbavancin treatment in staphylococcal osteoarticular infections [11,12]. Specifically, it was suggested that the maintenance of total dalbavancin concentrations of ≥4.02 or ≥8.04 mg/L over time could represent optimal pharmacodynamic (PD) efficacy thresholds against staphylococci exhibiting a MIC value up to the MIC90 or the EUCAST clinical breakpoint, respectively. Consequently, in a scenario of chronic vascular prosthetic infections requiring long-term treatment, TDM should be considered a valuable tool for personalizing dalbavancin therapy, given the wide interindividual PK variability and/or the need for eventual redosing.

Our aim was to describe our real-life experience with dalbavancin in difficult cardiovascular infections, with the support of TDM performed periodically in multidisciplinary long-term outpatient management.

## 2. Results

A total of 21 patients treated with at least a single dose of dalbavancin as sequential therapy for difficult-to-treat infections in our medical center were screened. Five patients were excluded because they had prosthetic osteoarticular infections or native endocarditis. Moreover, since our study intended to include patients who received at least two consecutive dalbavancin infusions 1 week apart, two patients were excluded, as they received a single infusion.

The final analysis included fourteen patients with cardiovascular prosthetic infections in the case series: eight cases of PVE (57.1%), seven PVGI (50%), and three of CEDI (21.4%); in four cases, there was an overlap of infection sites (28.6%).

Patient demographics and detailed clinical characteristics are summarized in Table 1 and Table 2. Eleven patients were male (78.6%), with a median age of 69.5 years (IQR 45–81 years).

*S. aureus* was the most isolated bacteria (5/14; 35.7%), with four cases of methicillin-sensitive *S. aureus* (4/5; 80%). There were three cases of infections caused by viridans streptococci (21.4%), two cases of coagulase-negative staphylococci (CoNS) (14.3%), one case of *E. faecalis* (7.1%)*,* one case of *Gemella morbillorum* (7.1%), and one case of *Corynebacterium striatum* (7.1%).

All patients were treated with empiric or targeted antibiotic therapy before dalbavancin treatment, with a median duration of 3 weeks (IQR 1–5 weeks). All patients had negative blood cultures before starting dalbavancin, and after the first dose, dalbavancin was continued as outpatient parental therapy.

In four cases, PVE and CEDI were complicated with peripheric embolization (4/10; 40%).

Only four patients achieved complete source control with implant removal (28.6%), and two patients underwent unsuccessful partial surgery (14.3%), while the remaining eight patients were not eligible for surgery due to comorbidities or surgical risk (57.1%).

**Figure 1:**
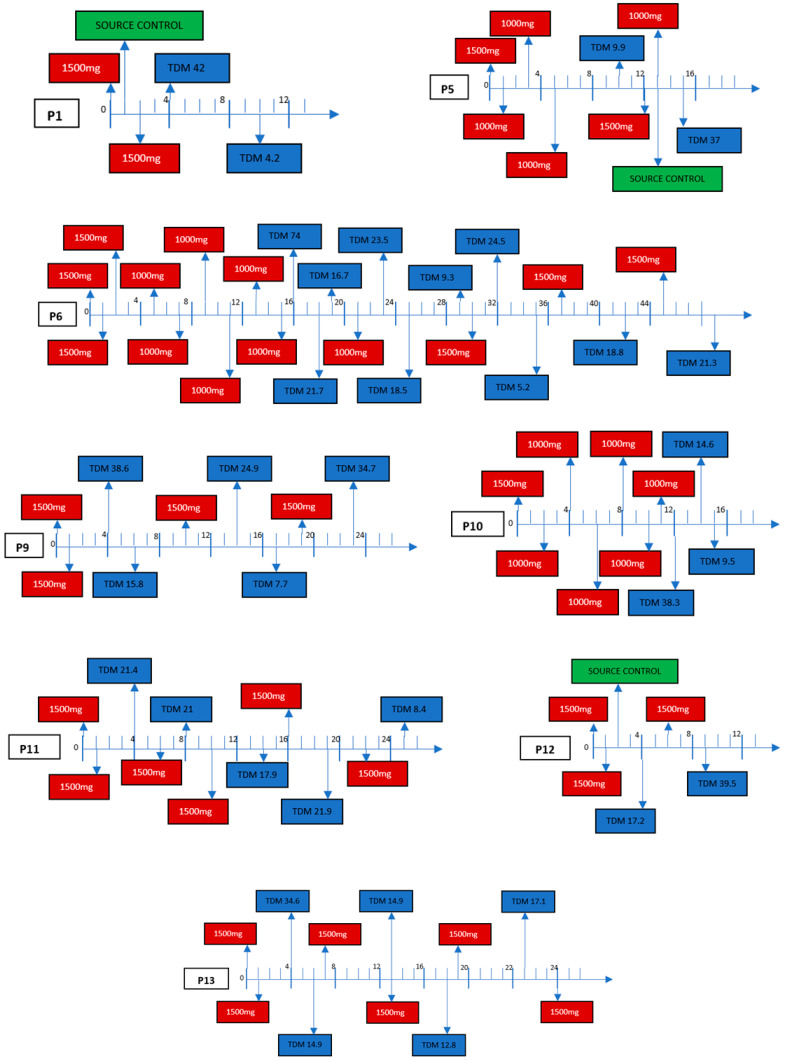
TDM-Guided Approach, Pharmacokinetic Variability (Each Line represents an interval of one week).

After the two 1500 mg dosing regimens 1 week apart (day 1 and 8), the additional dalbavancin doses were prescribed according to a TDM-guided strategy in nine patients (one patient still in ongoing therapy; Figure 1). Among patients managed according to a TDM-guided strategy, the timing of dalbavancin redosing ranged from 4 to 9 weeks between doses, mostly depending on renal function. The longest interval between infusions was observed in patient number 13, who suffered from chronic kidney failure, with an estimated glomerular function of approximately 30 mL/min (Figure 1). 

Overall, only 5 out of 34 dalbavancin TDM assessments were below the efficacy threshold concentrations of 8.04 mg/L (partly due to poor compliance of one of the patients). However, between 4.02 and 8.04 mg/L, showing good reliability of the TDM assessment method.

The median time between symptom onset and the end of treatment was 15 weeks (IQR 7–53), with significant differences by type of prosthetic infection. The median dalbavancin total duration was 8 weeks (IQR 1–45 weeks), with a median of 3.5 doses of dalbavancin infused (IQR 2–9) per patient.

The median length of follow-up was 65 weeks (IQR 23 to 144 weeks).

At the end of treatment, both clinical and microbiological successes were achieved in 10 cases (76.9%). Among the remaining cases, two patients changed antibiotic classes due to a lack of clinical improvement, one patient experienced microbiological relapse after dalbavancin interruption without developing drug resistance, and the last one is still on therapy. Clinical cure for patients who underwent the complete source control intervention was 100% (4/4), which reduced to 66.6% (6/9) in patients treated with suppressive antibiotic therapy without complete source control. Clinical success at the end of treatment was 75% (6/8) for patients with PVE, 83.3% (5/6) for PVGI, and 66% (2/3) for CEDI. Clinical cure according to causative pathogen was 60% for *S. aureus* (3/5), 100% for CoNS (2/2), 100% for *viridans Streptococcus* (3/3), 100% for *E. faecalis* (1/1), and 100% for *G. morbillorum* (1/1). The 18F-FDG PET/TC was available for nine patients, and in 6/9 (66.6%), radiological improvement with a marked SUV reduction was confirmed. Clinical success in the TDM-guided group was 87.5% (7/8), of which 50% (4/8) had implant retention versus 60% of patients treated without TDM (3/5). One patient is still in ongoing therapy.

Dalbavancin was well tolerated, only one patient developed a mild, self-healing rash that was infusion-related, and one patient affected by mild kidney insufficiency experienced a further slight deflection in renal function.

## 3. Discussion

Our case series confirms that dalbavancin could be an important therapeutic weapon in the treatment of cardiovascular infections, with an overall clinical and microbiological success rate of almost 77%. This rate is quite in line with those reported by Fazili T. et al., in a recently published review of 12 case series and 4 case reports evaluating dalbavancin efficacy in infective endocarditis in a total of 170 patients [10]. More recently, the Spanish multicenter study (EN-DALBACEN 2.0 Cohort) reported a 95.2% clinical success rate at 12 months of follow-up for 124 subjects with infective endocarditis, and 91.2% had undergone surgery prior to dalbavancin administration [13,14]. However, more than half of the patients included in these studies had native valve infections. On the contrary, in our case series, we selected only patients with prosthetic endocarditis, cardiac electronic implantable device infections, or prosthetic vascular graft infections, which are not included in the previous observational studies.

Cardiovascular prosthetic infections are a rare and serious complication of valve or vascular replacement, which are associated with high morbidity and mortality, significantly higher than native valve endocarditis [14,15]. These implants, steadily increasing, have surely improved quality of life and life expectancy, but they also represent a relevant risk factor for hospital-acquired infections, and their optimal diagnosis and treatment pose an emerging therapeutic challenge [14].

Although the best therapeutic option in prosthetic valve endocarditis is still debated, the recently published ESC guidelines on endocarditis recommend a surgical approach for early PVE and for PVE in high-risk subgroups identified by prognostic assessment (i.e., PVE complicated with heart failure, severe prosthetic dysfunction, abscess, or persistent fever), while patients with uncomplicated nonstaphylococcal late PVE could be managed conservatively. Moreover, the ESC guidelines point out that endocarditis in elderly patients may be burdened with increased surgical hesitation due to comorbidities, old age, and previous noncardiac and cardiac procedures, leading to less frequent performance of curative surgery and increased mortality compared with younger patients [16].

In our study, the rate of clinical success was 100% for patients who achieved complete source control. This emphasizes the crucial role of surgery in the proper management of cardiovascular prosthesis infections in line with the DALBACEN study [13]. However, although the best approach seems to be implant removal, older age, comorbidities, and general conditions may contraindicate this surgical approach. These patients, unable to undergo a surgical procedure, represent a major therapeutic challenge, and in this even more complex scenario, physicians must individualize treatment. Moreover, these infections are often associated with septic embolism and other peripheric localizations (36.4% in our case series), which makes the diagnostic and therapeutic pathway more difficult. Due to all these factors, patients with prosthetic cardiovascular infections may require long-term suppressive targeted antibiotic therapy [9,17].

The administration of parenteral dalbavancin, due to its good tolerability, safety profile, and favorable PK/PD characteristics, allowing prolonged intervals between consecutive doses, could be a promising option for these specific indications. Moreover, dalbavancin allows for a reduction in the number of hospitalization days, with a relevant impact on patient health and hospital resource management; indeed, in recent years, the treatment of patients outside the hospital has been increasingly in demand [18,19].

According to microbiological activity, our results showed a great prevalence of infections caused by Gram-positive cocci, in particular, *S. aureus* and CoNS [20], demonstrating the good clinical efficacy of dalbavancin. These data are in line with those recently published by Frazier et al., who demonstrated similar efficacy comparing the standard of care for treating *S. aureus* bacteria (treatment-related readmission rates within 30 days: 15% in the dalbavancin group vs. 22% in the SOC group, *p* = 0.484) [21]. Unfortunately, all patients received dalbavancin as sequential therapy, with a median duration of the previous regimen of 3 weeks, so the efficacy of dalbavancin as a primary regime in cardiovascular infections, alone or in combination regimens, must be investigated in future studies.

Another debated issue is the optimal treatment duration for prosthetic cardiovascular infection without implant removal. Fluorodeoxyglucose positron emission tomography (FDG-PET), as well as white blood cell (WBC) single photon emission computed tomography (SPECT)/CT (two nuclear imaging techniques considered alternatives in the new ESC guidelines) alone or in combination with computed tomographic angiography (CTA), can also help to diagnose and assess therapy duration in prosthetic valve endocarditis and vascular graft infections when deep sites are involved [15,16]. Nevertheless, evidence concerning the exact treatment duration is still lacking, and some evidence suggests that individualized, long-term antibiotic suppressive therapy, even exceeding 6 months or lifelong, appears to be the most effective strategy in patients unfit or unwilling to undergo high-risk cardiothoracic surgical interventions. 

In this difficult-to-treat scenario, our approach based on TDM-guided dalbavancin administration could be considered a valuable therapeutic strategy for ensuring the attainment of conservative PK/PD efficacy thresholds over time, thus possibly preventing potentially fatal complications and improving patient outcomes. Indeed, we observed a mildly higher proportion of clinical success when dalbavancin was used according to a TDM-guided approach (clinical success 87.5% vs. 60%, although not statistically significant, probably due to the small number of patients, so our results should be confirmed in a larger perspective cohort). This result suggests the relevance that adopting a TDM-guided approach may have in long-term dalbavancin therapy for providing a personalized treatment and for defining the proper time of redosing. This strategy, allowing maintenance of dalbavancin concentrations above conservative efficacy thresholds, could be associated with favorable clinical outcomes, as previously reported in the scenario of staphylococcal osteoarticular infections and suggested by a recent expert review panel [11,22]. 

Moreover, in patients with chronic kidney failure monitored with TDM, intervals between dalbavancin infusions were longer than in other patients due to the predominantly renal clearance of dalbavancin, as previously reported [1]. In this scenario, the use of TDM could also prevent unnecessary infusions. 

However, considering the wide interindividual PK variability, appropriate treatment duration should be guided ideally by means of real-time TDM coupled with Bayesian forecasting. Unfortunately, to date, therapeutic dose monitoring availability is limited to certain hospitals; nevertheless, considering pharmacokinetic variability between patients, the difficulty treating patients not eligible for complete source control, and the costs related to new drugs like dalbavancin, a periodic counseling service by hospitals performing TDM could be considered.

Our case series presents several limitations: (i) the limited sample size and monocentric nature of the study make the results underpowered; (ii) several primary regimens of antimicrobial therapies for at least 2 weeks prior to dalbavancin administration could overestimate dalbavancin efficacy; and (iii) heterogeneous follow-up after completion of treatment and no comparator treatment group make the results nongeneralizable. Considering these limitations, the overall rate of success must be interpreted with caution, and further larger studies are needed to confirm the additive value of TDM-guided dalbavancin administration for improving outcomes of cardiovascular prosthetic infections. 

## 4. Materials and Methods

This was a retrospective case series of consenting adult patients (≥18 years) treated with dalbavancin as sequential therapy for cardiovascular prosthetic infections. Data were collected from May 2019 to May 2023 at the Modena University Hospital, a tertiary care hospital in Northern Italy. Cardiovascular infections included PVE, CEDI, and PVGI.

For diagnosis, all patients underwent clinical, microbiological, and echocardiographic evaluation (transthoracic echocardiography and transesophageal echocardiography [TEE]). Major echocardiographic criteria included vegetation, abscess, pseudoaneurysm, intracardiac fistula, valvular perforation or aneurysm, and new partial dehiscence of prosthetic valve. In addition, according to clinical indication, computed tomography (CT) angiography or cardiac one and/or 18F-fluorodeoxyglucose (FDG)-positron emission tomography (PET) PET were also performed. 

As a case series, according to the Ethics Committee of Emilia-Romagna (Italy), such a project can simply benefit from a “waiver” by the EC chair, which exempts the ethics committee from evaluating the practice. Written informed consent was not required owing to the observational retrospective nature of the study.

Data collection included demographic information, Charlson Comorbidity Index (CCI), type and site of infection, microbiological isolation, prior antibiotic treatment, surgical procedure (performed or not, complete or partial), laboratory variables, and when available, radiological improvement (fluorodeoxyglucose positron emission tomography (FDG-PET) alone or in combination with computed tomographic angiography (CTA) to assess optimal duration of antibiotic treatment at follow-up).

Median duration of dalbavancin therapy, including dosage and number of administrations, was collected. Total duration of dalbavancin therapy was based on TDM assessment and the need for redosing [1].

Dalbavancin was prescribed two doses of 1500 mg one week apart on day 1 and day 8, infused over 2 h. For patients included after May 2021 who required long-term dalbavancin therapy, a TDM-guided approach was applied in order to maintain over time total dalbavancin concentrations above the conservative PK/PD efficacy thresholds of 8.04 mg/L and define the proper timing for dalbavancin redosing, as previously reported [12]. This conservative dalbavancin threshold ensures the attainment of optimal PK/PD efficacy against staphylococci exhibiting a MIC value up to the EUCAST clinical breakpoint of 0.125 mg/L [12]. A previous population PK model including 69 patients who received long-term treatment for subacute and chronic staphylococcal infections, including those affected by cardiovascular prosthetic infections, was used for estimating timing of dalbavancin redosing [1]. Long-term dalbavancin therapy was defined as continuation of antibiotic therapy for more than 4 weeks in challenging prosthetic infections. Timing for dalbavancin TDM assessments was arbitrarily selected by the treating physicians. Specifically, TDM of dalbavancin was assessed one or more times after completing the basic regimen of two 1500 mg doses 1 week apart on day 1 and 8, and timings were selected according to renal function of each patient, as previously reported [1].

Total dalbavancin plasma concentrations were measured at the Clinical Pharmacology Unit of the IRCCS Azienda Ospedaliero-Universitaria of Bologna by means of a validated liquid chromatography–tandem mass spectrometry analytic method, which was recently published [23].

Clinical evaluation was carried out at the end of treatment (EOT) and at the end of a six-month follow-up. Primary outcome was clinical cure, defined as resolution of all clinical signs and symptoms of infection, with or without radiological improvement. Secondary outcomes were (i) microbiological cure (sustained negativity of blood cultures), (ii) clinical relapse or microbiological recurrence with or without emergence of resistance to dalbavancin (persistence of signs and symptoms of infection, worsening of the clinical condition, new positive blood cultures with growth of the previously isolated pathogen), (iii) adverse events. Finally, for primary outcome, a comparison between patients treated with standard (the 1500 mg infusion regimen at day 1 and day 8, with subsequent 1000 mg administrations every 2 weeks) vs. TDM-guided approach was performed.

## 5. Conclusions

Our pivotal case series confirms the effective role of sequential therapy with dalbavancin among a selection of difficult-to-treat cardiovascular prosthesis infections with limited surgical indications. To the best of our knowledge, this study represents a larger case series of patients treated with suppressive, periodic TDM-guided dalbavancin courses of longer than 4 weeks. Our success rate seems to suggest that pharmacological data may be helpful for managing suppressive optimal treatment with long-acting lipoglycopeptides, guaranteeing greater effectiveness and safety. The strength of this promising data and the use of dalbavancin as a long-term suppressive treatment option for nonremovable prosthetic infections should be quickly evaluated in larger prospective studies to broaden therapeutic indications. 

## Figures and Tables

**Table 1:** Characteristics of the study population, treatments, and clinical outcomes.

Characteristics		
Variable	Total N = 14	
**Demographic characteristics and comorbidities**		
Age (years), *median IQR*	69.5	(45–81)
Male, *n (%)*	11	(78.6)
Charlson Comorbidity Index, *median IQR*	5	(0–10)
eGFR (mL/min/1.73 m^2^), *median IQR*	68	(32–122)
Albumin (g/dL), *median IQR*	3.55	(2.8–4.0)
**Type of infection, *n* (%)**		
Prosthetic valve endocarditis	8	(57.1)
Prosthetic vascular grafts infections	7	(50)
Multiple site infection	4	(28.6)
Cardiac implantable electronic devices infections	3	(21.4)
**Microbiological isolates, *n* (%)**		
*Staphylococcus aureus*	5	(35.7)
Methicillin-Sensitive *Staphylococcus aureus*	4	(28.6)
*Viridans streptococcus*	3	(21.4)
*Coagulase-Negative stafilococci*	2	(14.3)
*Enterococcus faecalis*	1	(7.1)
*Gemella morbillorum*	1	(7.1)
*Corynebacterium striatum*	1	(7.1)
Polymicrobial	1	(7.1)
Unknown	1	(7.1)
**Treatment characteristics**		
**Antibiotic first regimens, *n* (%)**		
Daptomycin	4	(28.6)
Ceftriaxone	4	(28.6)
Oxacillin	3	(21.4)
Rifampicin	3	(21.4)
Vancomycin	2	(14.3)
Doxycycline	1	(7.1)
Cefazoline	1	(7.1)
Gentamycin	1	(7.1)
Cefotaxime	1	(7.1)
Ampicillin	1	(7.1)
First line antibiotic median duration (weeks), *median IQR*	3	(1–5)
**Reason for dalbavancin administration, *n* (%)**		
Facilitate discharge	11	(78.6)
Adverse events	2	(14.3)
Poor compliance to therapy	1	(7.1)
**Median duration of dalbavancin therapy (weeks), *median IQR***	13	(5–49)
Median duration of dalbavancin therapy for indications, *median IQR*		
Cardiac implantable electronic devices infections (CEDIs)	25	(9–53)
Prosthetic vascular grafts infections (PVGI)	18.5	(13–46)
Prosthetic valve endocarditis (PVEs)	13	(7–46)
**Dalbavancin dosages and monitoring, *n* (%)**		
TDM	9/14	(64.3)
**Dalbavancin regimens, *n* (%)**		
1500 mg + 1500 mg, then according TDM	7	(50)
1500 mg +1500 mg	4	(28.6)
1500 mg + 1500 mg then 1000 mg every 14 d	3	(21.4)
**Clinical outcomes**		
Complete source control with implant removal, ***n* (%)**	4/14	(28.6)
Median follow-up (weeks), *median IQR*	65	(23–144)
Clinical cure at the EOT (*n* = 13)	10/13	(76.9)
Clinical cure at six months of follow-up (*n* = 12)	9/12	(75)
Clinical cure in TDM-guided group (*n* = 8)	7/8	(87.5)
Microbiological cure at the EOT (*n* = 13)	10/13	(76.9)
Microbiological relapse after treatment interruption (*n* = 13)	1/13	(8.3)
**Clinical cure by dalbavancin indications** at the EOT, ***n* (%)**		
Prosthetic valve endocarditis (*n* = 8)	6/8	(75)
Prosthetic vascular grafts infections (*n* = 6)	5/6	(83.3)
Cardiac implantable electronic devices infections (*n* = 3)	2/3	(66.6)
**Adverse effect reactions**		
Rash, *n* (%)	1	(7.1)
Impaired renal function, *n* (%)	1	(7.1)

Abbreviations: IQR, interquartile range; eGFR, estimated glomerular filtration calculated by means of the CKD-EPI formula; TDM, Therapeutic Drug Monitoring; EOT, end of treatment.

**Table 2:** Detailed characteristics of the patients included in the case series.

Patient	Type of Infection	Aetiology	First Regimen	First Regimen Duration	Dalbavancin Regimen	Dalbavancin Duration	CCI	Prosthetic Removal	TDM	Clinical Success End of Treatment (Total Weeks from Beginning of Antibiotic Therapy)	Clinical Success Six Months Follow-Up	Follow-Up (Weeks)	Death (Weeks from End of Treatment)
1 (62 y)	CEDI infection	MSSA	Oxacillin	3 weeks	1500 mg + 1500 mg	6 weeks	4	Yes	Yes	Yes (9 w)	Yes	65 w	No
2 (80 y)	Periprosthetic aortic abscess at the mitro-aortic junction + thoracic aortic endoprosthesis infection in myelofibrosis (PVE + PVGI)	Unknown	Doxycycline + rifampin, then daptomycin + ceftriaxone	3 weeks	1500 mg + 1500 mg then 1000 mg every 14 d	13 weeks	9	No	No	No (16 w)	No	-	Yes (47 w)
3 (45 y)	Periprosthetic aortic abscess (PVE)	*Gemella morbillorum*	Vancomycin + gentamycin then daptomycin + gentamycin	3 weeks	1500 mg + 1500 mg	6 weeks	1	Yes	No	Yes (9 w)	Yes	144 w	No
4 (80 y)	Prosthetic aortic endocarditis (PVE)	*Enterococcus faecalis*	Ceftriaxone + ampicillin then daptomycin + ceftriaxone	4 weeks	1500 mg + 1500 mg	6 weeks	7	No	No	Yes (10 w)	Yes	46 w	Yes (46 w)
5 (56 y)	Aortic endoprosthesis infection (PVGI)	*Staphylococcus capitis*	Daptomycin + ceftriaxone then oxacillin + rifampin	3 weeks	1500 mg + 1500 mg then 1000 mg every 14 d	17 weeks	3	Yes	Yes	Yes (20 w)	Yes	78 w	No
6 (50 y)	CEDI infection with peripheric embolization and spondylodiscitis	MSSA	Daptomycin + cefazolin then daptomycin then teicoplanin	4 weeks	1500 mg + 1500 mg then 1000 mg every 14 d, then according TDM	49 weeks	4	Partial	Yes	No (53 w)	No	-	No
7 (80 y)	Prosthetic aortic endocarditis with peripheric embolization with stroke, splenic and intestinal infarctions (PVE)	MSSA	Cefazolin + rifampin	4 weeks	1500 mg + 1500 mg, missing data concerning timing additional doses	8 weeks	10	No	No	No (12 w)	No	-	Yes (21 w)
8 (69 y)	Prosthetic aortic endocarditis (PVE)	*Streptococcus mitis/oralis*	Daptomycin + ceftriaxone	1 week	1500 mg + 1500 mg	6 weeks	7	No	No	Yes (7 w)	Yes	91 w	Yes (91 w)
9 (81 y)	Prosthetic aortic endocarditis and CEDI infection, spondylodiscitis (PVE + CEDI infection)	*Staphylococcus lugdunensis*	Oxacillin	1 weeks	1500 mg + 1500 mg then according TDM	24 weeks	8	No	Yes	Yes (25 w)	Yes	44 w	Yes (44 w)
10 (51 y)	Prosthetic aortic endocarditis with splenic abscess and spondylodiscitis + ascending aorta endoprosthesis infection (PVE + PVGI)	*Streptococcus sanguinis*	Daptomycin + ceftriaxone then daptomycin + ampicillin	4 weeks	1500 mg + 1500 mg then 1000 mg every 14d	14 weeks	0	No	Yes	Yes (18 w)	Yes	83 w	No
11 (70 y)	Axillo-femoral bypass infection (PVGI)	MRSA	Vancomycin	3 weeks	1500 mg + 1500 mg then according TDM	26 weeks	5	No	Yes	Yes (29 w)	Yes	23 w	No
12 (56 y)	Aortic endoprosthesis infection (PVGI)	MSSA + *Acinetobacter baumanniii*	Oxacillin + rifampin then association with cotrimoxazole	3 weeks	1500 mg + 1500 mg then according TDM	11 weeks	3	Yes	Yes	Yes (14 w)	Yes	38 w	No
13 (78 y)	Prosthetic aortic endocarditis + ascending aorta endoprosthesis infection (PVE + PVGI)	*Sreptococcus pasteurianus*	Cefotaxime then ampicillin	6 weeks	1500 mg + 1500 mg then according TDM	41 weeks	3	No	Yes	Yes (47 w)	-	-	No
14 (72 y)	Endovascular graft infection (PVGI)	*C. striatum*	Teicoplanin then linezolid	5 weeks	1500 mg + 1500 mg then according TDM	ongoing	6	Partial	Yes	-	-	-	No

Abbreviations: CEDI, cardiac implantable electronic devices infections; PVE, Prosthetic valve endocarditis; PVGI, Prosthetic vascular graft infection; MSSA, Methicillin-sensitive Staphylococcus aureus; MRSA, Methicillin-resistant Staphylococcus aureus; CCI, Charlson Comorbidity Index; TDM, therapeutic drug monitoring.

## Data Availability

All useful data used to write this article are included in tables and figure reproduced above.
